# Extracting Sensory Preferability from Motor Streams

**DOI:** 10.3390/s25072087

**Published:** 2025-03-26

**Authors:** Vilelmini Kalampratsidou

**Affiliations:** Department of Product and System Design, Aegean University, 84100 Ermoupolis, Syros, Cyclades, Greece; vilelmini.kalabratsidou@gmail.com

**Keywords:** movement analysis, sensory–motor system, afferent and efferent signals, reafference principle, contextual preferability

## Abstract

(1) Background: Based on the reafference principle, our system creates an efferent signal copy to distinguish external inputs from our activities in the afferent signal. According to this principle, sensory and motor information from the outside world travel together from the periphery to the brain. (2) Methods: This work introduces signal processing methods that extract contextual sensory preferences from motor streams. Speed and acceleration data were collected as participants walked under different conditions: in silence (with open and closed eyes), while listening to two different songs (each with open and closed eyes), and finally while walking to their favorite song. Ten individuals completed a total of seven conditions. (3) Results: Variations in the walking patterns of each participant were identified, revealing the sensory inputs they perceived. The results also indicated the audio and visual conditions that optimized the participant’s sensory–motor system performance. (4) Conclusions: The outcomes suggest that we can extract from motor stream particulars that go beyond an individual’s movement qualities and toward the contextual sensory inputs accompanying the movement data, even when participants execute the very same task of walking.

## 1. Introduction

Human movement recording is increasingly becoming a standard practice across various scientific domains. There are numerous technologies, which vary from highly costly and novel motion-capture systems to reasonably priced, watch-size inertial measurement units (IMUs) and sensors embedded in everyday devices such as smartphones and smartwatches, including ‘stepometers’ (also known as step counters). Thanks to these technologies, position data, linear and angular acceleration, speed, and jerk are among the motion streams that can be recorded. Their applications span numerous fields such as medicine, biomechanics, robotics, the army, interactive arts, animation, and filmmaking, among others [1]. Notably, their use has been expanded to include studies on the motor patterns of other animals [2,3,4].

Given the wide use of these technologies, there is a broad range of methods used for analyzing and processing motor signals, as well as a wide range of perspectives on investigating movements. In biomechanics, researchers are interested in studying the healthiest ways to move and strategies for quick recovery after an injury. In robotics, scientists attempt to make different robotic structures move like living organisms. The interactive arts community focuses on employing human movement, typically in real-time, to promote interactivity and stimulate learning. In animation and filmmaking, the goal is to translate physical movement into virtual work. Meanwhile, the field of movement neuroscience continues to work on creating and refining theories of how the peripheral nervous system (PNS) and central nervous system (CNS) coordinate to generate movement.

In movement neuroscience and human physiology, when moving, an outgoing movement signal, called an efferent signal, is sent from the CNS to the periphery. The sensory input received from receptors is called afference; it is blended with reafference, which is the sensory input generated by our own actions. The two channels of the afferent and efferent signals describe the transfer of information from the CNS to the PNS and vice versa.

### 1.1. Reafference Principle and Sensory Input in Motor Stream

The reafference principle is a fundamental concept used to understand the interactions between efferent and afferent channels. Based on this, when an efferent copy is generated, a duplicate, known as the efferent copy, is created. In this way, the nervous system can distinguish between exafference (sensory input from external stimuli) and reafference (sensory feedback from a movement that is self-generated). Therefore, the nervous system is able to differentiate between self-initiated actions and external inputs.

The principle of reafference was developed to explain the interaction between outgoing and incoming signals in directing and coordinating movement. It also accounts for the phenomenon where stimuli caused by self-initiated actions do not disrupt perceptual constancy [5]. Additionally, it has been instrumental in studying the adaptive control of a movement task. In the principle of reafference by [6], it was stated that “Voluntary movements show themselves to be dependent on the returning stream of afference which they themselves cause”. In addition, empirical evidence concerning the influences of past voluntary motions on future intended actions has given rise to the notion of internal models for action (IMAs) [7,8,9,10]. Thus, it has been theorized that the brain creates internal models of its own self-generated dynamics and adapts them, adapting from one situation to another.

This principle has greatly advanced research on motor learning. However, one persistent challenge in the field involves how to computationally disentangle the sensory from the motor components from the afferent stream. Indeed, the motor streams collected from any equipment, such as motion-capture systems and IMUs, inherently contain external sensory information.

It is also interesting that anatomically, motor afferent and motor efferent signals travel through different fibers [11]. Functionally, however, they are integrated and dynamically change from moment to moment and context to context. This dynamic relationship between the two types of signals, along with the fact that motion-recording technology captures signals enhanced with external stimuli, raises the following compelling questions:

“Can we detect sensory variations within motor streams?”

“Is it possible to separate sensory and motor components automatically?”

“Could researchers develop methods to extract sensory information from continuous motor data?”

Addressing these questions could lead to advances in our understanding of motor control and the sensory contributions embedded in motor streams.

### 1.2. Interfaces in Need of “Understanding” the Preferability of Our Peripheral Nervous System

The importance of detecting sensory preferences through the motor stream could be particularly useful for interfaces designed to restore or enhance the wellness of our peripheral system. Many interfaces aim to understand and adapt to human motor needs, often becoming an extension of the human motor control system. Examples include exoskeletons and limp extensions traditionally controlled by an external device or directly by the brain [12,13,14]. Exoskeletons are often used for rehabilitation purposes, for instance, the robot described in [15] is made for arm therapy applicable to the clinical setting. A similar example is demonstrated in [16], which describes a system designed to assist in repetitive therapy tasks related to activities of daily living.

In therapy and rehabilitation, identifying external sensory conditions that enhance a patient’s PNS performance and accelerate the learning process is indeed vital. Such knowledge could significantly improve the effectiveness of the therapy and accelerate the rehabilitation process.

Co-adaptive interfaces also present exciting opportunities by relating movement with external feedback, such as audio [17,18], and based on the participant’s performance, these systems adapt the audio feedback. One key advantage of such interfaces is their ability to study the factors that influence and accelerate the process of adaptation. A second key advantage—when they are designed to work in real-time—is the automatic adaptation of the computational system to the personalized preferred conditions of the participant, rather than relying on predefined settings. This opens up new research opportunities, enabling the study of dynamic, hands-off interactions between users and interfaces.

Other real-time interfaces connect physiological information, such as heart rate, with external stimuli, such as song tempo [19,20,21]. These studies investigate how augmenting bodily information—such as heart rate—can induce detectable changes in the human system. While these two interface examples primarily rely on non-movement bodily signals, the study of movement could provide valuable insights. Based on the reafference principle, sensory and motor information travel together in our PNS.

These studies emphasize the need for computational techniques that can detect sensory preferability, i.e., the conditions that best encourage the sensorimotor system to operate more efficiently and healthily. These techniques, when combined with co-adaptive interfaces, could create powerful new research tools for studying and optimizing the interaction between humans and technology.

### 1.3. Baseline Research

To reach the point of creating co-adaptive interfaces, a series of method development studies were conducted in motor streams and other bodily signals (such as body temperature and ECG signals), as presented in [22].

A basic data structure used in all baseline studies is called micro-movement spikes (MMS). Previous research of MMS [23,24,25] indicates that the MMS structure enables the study of the spatiotemporal features of signal fluctuations by investigating the normalized peaks (or maxima) of the unfolding motor streams. These fluctuations reflect changes in the context and sources of sensory guidance, among other aspects of the sensory–motor signals. In [26,27], the MMS structure was employed to examine how walking is differentiated in populations with autism and children with Phelan–McDermid syndrome. Specifically, the stochastic signatures of kinematic parameters were estimated to interpret the dynamic motor patterns of the human motor system when walking and, hence, investigate differences in the “noise” parameter of the Gamma distribution (also known as the scale) between autistic and non-autistic motor systems. Next, the MMS structure of the acceleration stream was studied in relation to the body temperature, building a new data structure that enabled the investigation of the moment-to-moment fluctuations of both signals [28]. Later, the MMS structure was applied to ECG data to investigate how the stochastic signatures of the heart activity shift when dancing [20]. Apart from analysis in the time domain, the MMS structure was also used in the frequency domain, where a wide range of parameters is available for study. For example, in [29], the authors investigated the speed and bending parameters in the time domain employing MMS, which were then transformed into the frequency domain to estimate the maximum coherence value and their corresponding phases. This analysis led to the examination of the inter- and intra-connections of two bodies partnering in ballet.

An early discovery concerning the sensory information carried in the motor stream was revealed in Ref. [30]. The outcomes indicated that the rhythm of music impacts the temporal features of bodily signals. Specifically, it revealed the entrainment of human speed and heart activity in the presence of music.

### 1.4. Aims and Contributions

In this study, the MMS structure was combined with Gamma analysis to investigate the motor streams—speed and acceleration data—of 20 body parts from 10 participants performing the physical task of walking under various audiovisual conditions. The Gamma continuous process was applied within the framework of Poisson random processes, allowing the data to be analyzed as if it were real-time, despite being pre-recorded.

To enrich the analysis, Gamma parameters were accumulated, and their slope values and the ratio defined by the stochastic signature location on the Gamma plane were extracted. This process created new parameter spaces that facilitated the clustering of kinematic data based on sensory input. Consequently, this approach enabled the automatic detection of preferred sensory inputs from the afferent motor signals extracted from the motor stream.

This method of information retrieval seems to be connected to the principle of reafference (as presented in Section 1.1), which states that the reafferent signal traveling in the periphery includes external sensory information. The methods were applied both in speed and acceleration streams to confirm the outcomes and to research further possibilities of information retrieval.

In this paper, Section 2 presents all the steps—from the estimation of the speed and acceleration stream to the construction of the MMS and the creation of the new parameter spaces. Section 3 displays the results of the speed data. The outcomes of the acceleration data are shown in the Supplementary Material. Section 4 discusses the findings in relation to the principle of reafference and the possible applications of the presented methods across different fields. And lastly, Section 5 summarizes the work.

## 2. Experimental and Computational Methods

### 2.1. Participants

Ten people (three males and seven females) participated in the study. They ranged from 12 to 45 years old. All participants signed the consent form approved by the Institutional Review Board (IRB) of Rutgers University, in compliance with the Helsinki Act.

### 2.2. Experimental Task

Participants were asked to walk naturally under different contexts. First, they had to walk in silence with open eyes, then in silence with closed eyes. In the next four conditions, they had to walk while listening to two pre-determined songs (“Back it up” by Caro Emerald and “Experience” by Ludovico Einaudi), first with open eyes (conditions three and four) and then with closed eyes (conditions five and six). In the seventh condition, they walked with open eyes while listening to their favorite song; see Table 1.

In all conditions, participants were instructed to walk naturally and to let the music influence their movements. They were even encouraged to dance to the music while walking. Yet, all participants simply walked.

#### Sensory Stimuli

The different types of music were used to create different conditions of sensorimotor preferability. Two were pre-selected and played to all participants. The third one was the favorite song of each participant.

The two pre-selected songs—common to all participants—had different features. The song “Back it up” by Caro Emerald is a very rhythmic pop song with a clear and stable tempo. The song “Experience” by Ludovico Einaudi is classical with an escalating structure. The favorite song was used to evaluate whether participants’ music choices were related to the sensory–motor preferences extracted from the presented methods.

Audio feature extraction and autocorrelation analyses were performed to characterize the auditory sensory stimuli in [30].

### 2.3. Instrumentation

**Figure 1:**
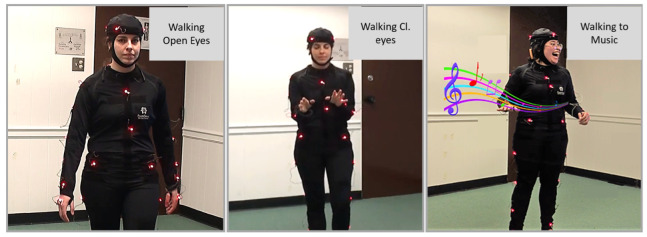
Moments from three different conditions of the experimental tasks: “Walking”, which was executed with open eyes, “Walking Cl. Eyes” where the participant used her hand to make sure she would not step over anything, and “Music 1” where she walked with open eyes while listening to music.

**Figure 2:**
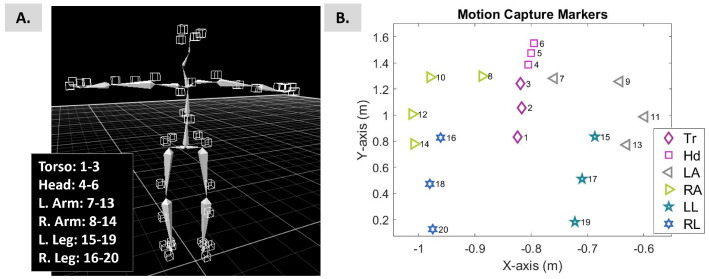
(**A**) The 3D skeleton of the participant in a T-pose was built using Recap2 V2.0 Software developed by PhaseSpace. T-pose is required by the specific software to estimate the bone lengths, develop the participant’s skeleton, and enable the streaming and saving of the participant’s bone data. (**B**) The X-Y-Z positional data of the bones of the participant were loaded in MATLAB R2018B after streaming was enabled. The participant stood in a neutral pose.

An 8-camera motion-capture system named PhaseSpace Impulse (480 Hz San Leandro, CA, USA) was used to record bodily positions and movement. A total of 38 active light-emitting diodes (LEDs) were mounted on a suit (Figure 1) to build the participant’s skeleton (Figure 2A).

As will be more analytically explained later, the movement data types used in this study were speed and acceleration. Therefore, the outcomes of this study are not restricted to the use of a motion-capture system but could be expanded to wearable IMUs or other sensors that enable the direct extraction or estimation of speed or acceleration data.

### 2.4. Data Processing

#### 2.4.1. Extraction and Computation of Motor Stream

**Figure 3:**
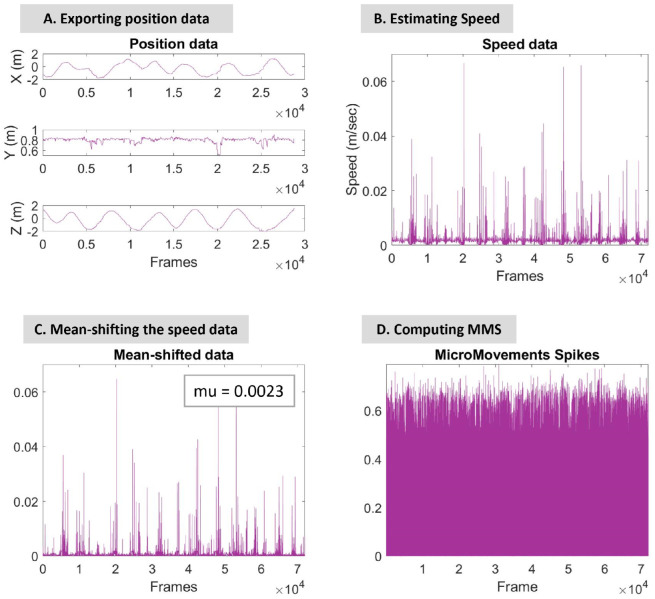
Signal processing steps and MMS estimation. Step (**A**) Raw positional data of the hip were exported using Recap Software from PhaseSpace Motion Capture. The figure displays the variability of the X, Y, and Z axis position data over the number of frames. Step (**B**) The estimated speed of the specific body part. Step (**C**) The speed data mean-shifted by 0.0023. Step (**D**) MMS computation.

Using Recap software (by PhaseSpace Motion Capture), the positional data of 20 bones (4 for each arm, 3 for each leg, head, and torso) were exported; see Figure 2B. The sampling rate was 480 Hz. For each stream of positional data, the speed and acceleration were estimated and used in the analysis below; see Figure 3A.

The main document presents the results from the speed data analysis. The outcomes of the acceleration data are shown in the Supplementary Material.

To estimate the speed, the 1st derivative for each X, Y, and Z axis (Figure 3A) was estimated and then the norm was calculated using the following equation: $speed=\sqrt{v_{x}^{2}+v_{y}^{2}+v_{z}^{2}}$. Similarly, the acceleration was calculated by the 2nd derivative for each X, Y, and Z axis and then the norm was estimated using the following equation: $accel=\sqrt{a_{x}^{2}+a_{y}^{2}+a_{z}^{2}}$.

Next, the signal was mean-shifted, as demonstrated in Figure 3C, where the mean value of the example hip-speed data is mu=0.0023.

#### 2.4.2. Micro-Movement Spikes

Micro-movement spikes (MMS) (Figure 3D) are the peaks of the signal normalized to conserve the range of amplitude changes and the time of the peaks. As a result, they are real-valued numbers ranging from 0 to 1. Normalization is attained by dividing each peak value by the sum of the peak value and the average value between the two adjacent local minima [23], as shown in the following equation:

(1) $$LocalPeak_{normalized}=\frac{LocalPeak}{LocalPeak+LocalAverage_{mintomin}}$$

This standardization scales the data while accounting for the allometric effect, which appears in kinematic data due to the anatomical differences across participants (e.g., height, size) [31,32].

The micro-movement spikes (MMS) are estimated for each kinematic signal, speed (Figure 3D), and acceleration.

#### 2.4.3. Micro-Movement Spikes Represented by Continuous Gamma Process

The micro-movement spikes (MMS) of the kinematic streams are best described by the Gamma family of distributions. This was empirically determined by the maximum likelihood estimation (MLE), which was utilized to select the best continuous family of probability distribution functions (PDFs) that fit the frequency histograms of their peaks.

In the Gamma family of distributions, a random variable, *X*, distributed with shape *a* and scale *b*, is denoted by X~Γ(a,b)=Gamma(a,b), with a probability density function:

(2) $$f\left(x,a,b\right)=\frac{x^{a-1}e^{-x/b}}{b^{a}\Gamma \left(a\right)},\;\mathrm{for}\;x>0\;\mathrm{and}\;a,b>0$$

The Gamma mean is denoted by μ=ab and the variance is denoted by $\sigma =ab^{2}$, where the noise-to-signal ratio, $NSR=\frac{\sigma }{\mu }=b$, is also known as the scale parameter.

Previous research has also indicated that the Gamma family of distributions best characterizes these data types [23,28,33,34].

Thus, in the present study, a continuous random process was employed under the general rubric of Poisson random processes (commonly used in electrophysiology for the analyses of cortical spike trains) to represent MMS; and a continuous Gamma process was adopted to represent continuously processed blocks of these fluctuations using a relaxed independent identically distributed (IID) assumption.

**Figure 4:**
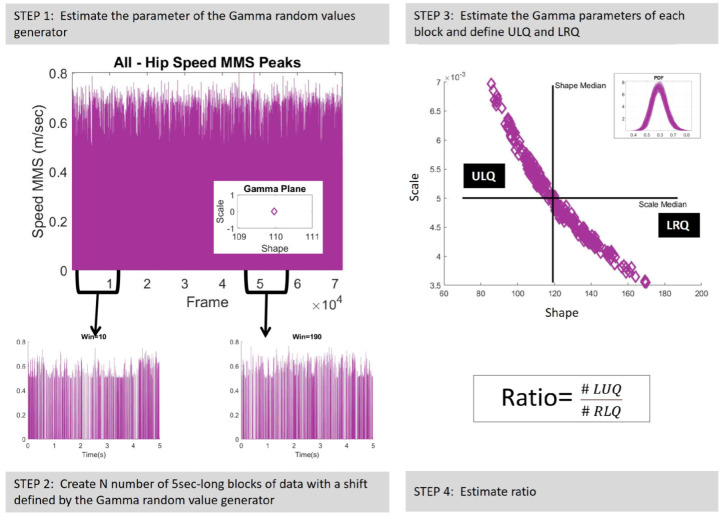
Continuous random process. Step 1. Estimate the Gamma parameters. Displayed in the inset—characterize the MMS of the hip speed under a specific condition to create a Gamma random value generator. Step 2. Create a 5 s ($5*sampling_{rate}$) window, which is randomly shifted by a number returned by the Gamma random value generator. Step 3. Gamma parameter estimation for each block of data. Step 4. Estimate the shape and scale medians to define the ULQ and LRQ; and compute the quadrant participation ratio, which is equal to the number of signatures in the ULQ divided by the number of signatures in the LRQ.

The block size was set equal to 5 seconds (5 times the sampling rate of the data, 480 Hz). This block size ensured enough peaks to perform MLE with tight confidence regions (95%). The shift step was set to a random Gamma value generated by the generator developed in Step 1, which was updated for each new window. Thus, N number of 5 sec long blocks were created (Step 2, Figure 4). Next, the Gamma parameters of each block were estimated. Figure 4, Step 3, presents the shape and scale parameters of the Gamma plane in linear space and the inset figure displays the probability density functions (PDFs) of the corresponding Gamma signatures. Along with the shape and scale parameters, the four moments, that is, the mean (as described in Equation (2)), variance (as described in Equation (2)), skewness, and kurtosis were also investigated.

The Gamma parameters were later used to define the upper-left quadrant (ULQ) and lower-right quadrant (LRQ) by extracting the median values of the shape and scale parameters. Finally, the ratio of the number of Gamma signatures laying on ULQ over the number of Gamma signatures laying on LRQ was estimated (Step 4, Figure 4).

#### 2.4.4. Tracking NSR and Predictive Regimes

**Figure 5:**
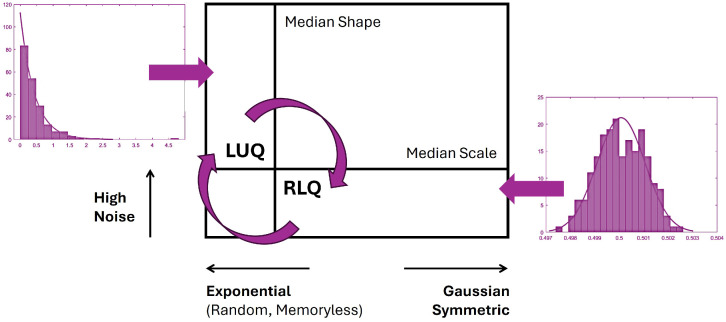
Empirically informed interpretation of the Gamma parameter plane to further help statistical inference of the ULQ/LRQ ratio metric. Transitions across these quadrants reveal different distributions with different shapes and scale parameters that help track the frequency of visits to each quadrant through time, as also explained in [20,35].

The median statistic was employed to separate signatures with low NSR (equal to the scale parameter) and high symmetry (described by high shape values) from points of high NSR and low symmetry (high skewness) that are located in the ULQ. Signatures that are located in the LRQ tend to the Gaussian range of the Gamma family. Whereas, signatures that are located in the ULQ tend to the exponential range of the Gamma family. Figure 5 illustrates these two antithetic cases.

Previous empirically informed models have shown that nervous systems with pathologies are well-characterized by the exponential case [23,25]; typical populations are well-characterized by intermediate skewed ranges with variable NSR, and athletes and ballet dancers are well-characterized by the symmetric and low NSR cases [28,29].

Therefore, the ratio LUQ/RLQ, which is defined by the number of points in each quadrant, provides information about the NSR and the predictability of the nervous system. As such, a ratio that is less than 1 and closer to 0 characterizes a predictive system. In contrast, a ratio that is higher than 1 describes a more noisy and less predictive motor system.

#### 2.4.5. Euclidean Distance of the Extreme Signatures

The Euclidean distance of the two logarithmic Gamma line extreme signatures was estimated as a tool that indicates the size of the shift from the noisiest to the least noisy and most predictive performance that a specific nervous system can achieve. Among individuals with no motor impairment, this metric is an indication of the learning effort that was put in by the body part of the nervous system but does not necessarily characterize the best outcome or the overall performance.

#### 2.4.6. Rate of Change in Stochastic Transitions

The log–log representation of the Gamma parameter (shape and scale) plane is then estimated. A line fitting $f\left(x\right)=nx^{m}$ in linear representation captures the rate of change of the block-to-block signature transitions. This information lies in the slope of the log–log representation, which is equal to the exponent of the linear representation.

The fitting error (delta, Δ) is also measured by the projection of each point to the fitting line of the log–log scale.

Slopes with absolute values larger than 1 indicate a faster rate of change toward the LRQ. Thus, a desirable state along with a ratio below 1 is when the slope is above 1.

#### 2.4.7. Cumulative Information

The Gamma distribution is an exponential family and represents an additive random process. As such, in this work, the values of the log-shape and log-scale parameters are accumulated across the kinematic chain. Specifically, after estimating the windows of each body part following the continuous Gamma process, as demonstrated in Figure 4, we were able to accumulate the log-shape and log-scale parameters in the order of the kinematic chain of the first windows, repeat this with the second windows, and so on. In this way, we were able to estimate the cumulative shifts of the log-shape and log-scale parameters of the kinematic chain from block-to-block, or throughout time.

#### 2.4.8. Statistical Significance

The non-parametric one-way analysis of variance (ANOVA) Kruskal–Wallis test was employed in two points of the study along with the “multcompare” function in MATLAB. Multcompare performs a multiple comparison using the outcomes of the Kruskal–Wallis test to determine which estimates are significantly different.

Hence, the Kruskal–Wallis test was first used to examine the Euclidean distance between the two extreme signatures of the log–log Gamma plane. Next, it was applied to the four-moment parameters of each participant. The goal was to differentiate the general effects of experimental conditions (columns) over body parts (rows).

Another test used to examine the significance of the outcomes was the “ranksum” Wilcoxon rank-sum test for equal medians. This test was employed to compare the outcomes of the slope values—the first involved conditions that included music versus the condition with no music and the second involved conditions where walking was conducted with open eyes versus closed eyes. The same comparisons were applied to the ratio values.

## 3. Results

### 3.1. Investigating the Gamma Stochastic Signatures in Logarithmic Space

**Figure 6:**
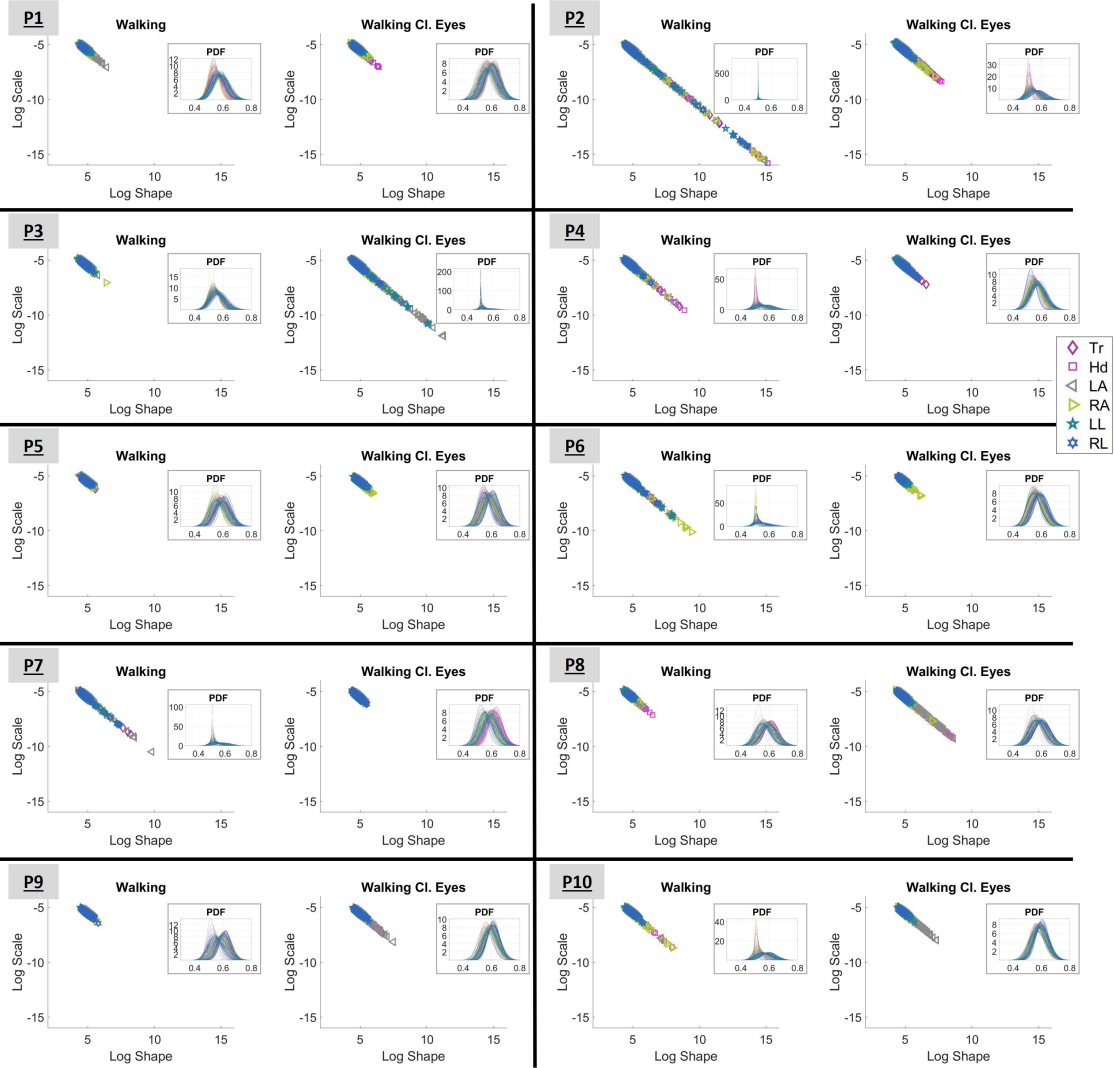
Log–log representation of the shape and scale parameters of the Gamma distribution for the conditions “Walking” and “Walking with closed eyes”. The demonstrated plots correspond to all participants, from P1 to P10. The color-mapped markers correspond to different body parts (grouped as presented in Figure 2). Tr stands for torso; Hd for head; LA for left arm; RA for right arm; LL for left leg; and RL for right leg. The insets highlight the PDFs of the presented Gamma signatures. The X-axis in the inset represents MMS (the normalized peaks of the speed) and the Y-axis represents probability density.

The log–log representation of the Gamma process analysis is presented in Figure 6. The displayed plots correspond to conditions “Walking” (with open eyes) and “Walking with Closed Eyes” for all participants (P1 to P10). Each stochastic signature is described by the log-shape and log-scale parameters of Gamma distribution (as explained by Equation (2)) and corresponds to the MMS of the speed data of a body part (20 in total) during a block of data, as presented in Section 2.4.3. A total of 182 blocks were generated for each body part. The outcomes of the acceleration data are presented in Supplementary Figure S1.

Observing Figure 6, it is interesting to note that the range within which the signatures lie differs from condition to condition and from participant to participant. All displayed signatures seem to start around the point (4,−5); however, the lower right end of the “line” varies.

As explained in Section 2.4.4, signatures that lay on the LRQ characterize systems with lower NSR and higher symmetry and, therefore, predictability. As such, based on Figure 6, P2, P4, P6, and P7 seem to perform better when “Walking” (with open eyes). In contrast, P3, P8, and P9 have more predictable behavior when “Walking Cl.Eyes”. For the rest of the participants (P1, P5, and P10), no difference can be noted visually.

To better investigate this, the Euclidean distance between the two extreme signatures was estimated. Table 2 demonstrates the results of all participants and conditions. Here, it is easier to investigate the differences between conditions “Walking” and “Walking Cl.Eyes” for P1, P5, P10. Further, it can be noted that P9, P6, P4, and P5 have the most conditions with short distances (below 2—highlighted in red) and the lowest means (in the order written). In contrast, P2, P1, and P3 have no short distances, and they hold the highest means of Euclidean distances (in the order written). The estimated Euclidean distances of the acceleration data are presented in Supplementary Table S1.

**Figure 7:**
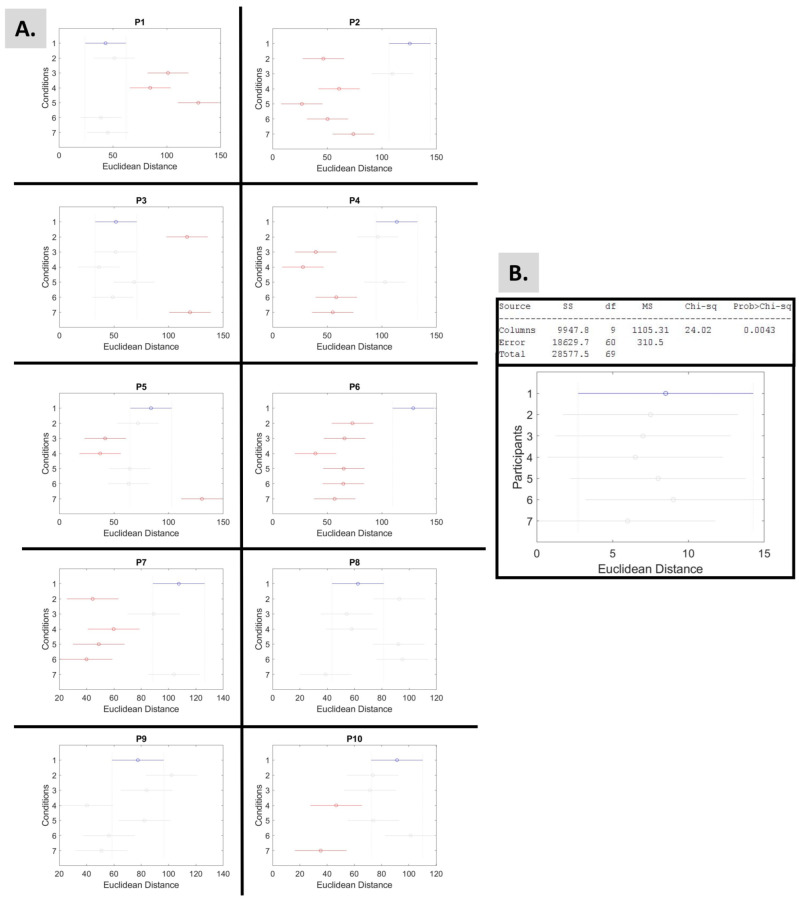
Results from the non-parametric Kruskal–Wallis and multiple comparison post hoc tests applied to the Euclidean distance findings. In each graph, the selected group (colored blue) is significantly different from red groups and non-significantly different from grey groups. By default, the selected group is condition 1. (**A**) The test aimed to investigate the effects of the experimental conditions (columns) over the Euclidean distances of the body parts (row) of each participant. The *p*-values of the findings are presented in Table 3. (**B**) The test aimed to investigate differences among participants (columns) over the Euclidean distances of the conditions (row).

Next, the Kruskal–Wallis test, which performs a non-parametric one-way ANOVA, revealed that the Euclidean distances of each participant (as shown in Table 2) demonstrate statistical significance (Figure 7B). Moreover, the same test was used to investigate the effects of the experimental conditions (columns) over the Euclidean distances of the body parts (row) of each participant. The outcomes are shown in Figure 7 and Table 3, demonstrating statistical significance.

Based on the criteria of the Euclidean distance, or length of the logarithmic Gamma line, P1 performs better during condition 5, “Music Cl. Eyes 1”; P2 during “Walking”; P3 during “Walking Cl. Eyes”; P5 during “Fav. Music”; P6 during “Walking”; P7 during “Walking”; P9 during “Walking Cl. Eyes”; and P10 during “Music Cl. Eyes 2”, as Figure 7A indicates.

### 3.2. Observing Variations Within the Kinematic Chain of Each Participant

Another metric used to investigate the Gamma plane was the slope, as presented in Section 2.4.6. This metric allowed us to investigate the slope variations within the kinematic chain of each participant.

**Figure 8:**
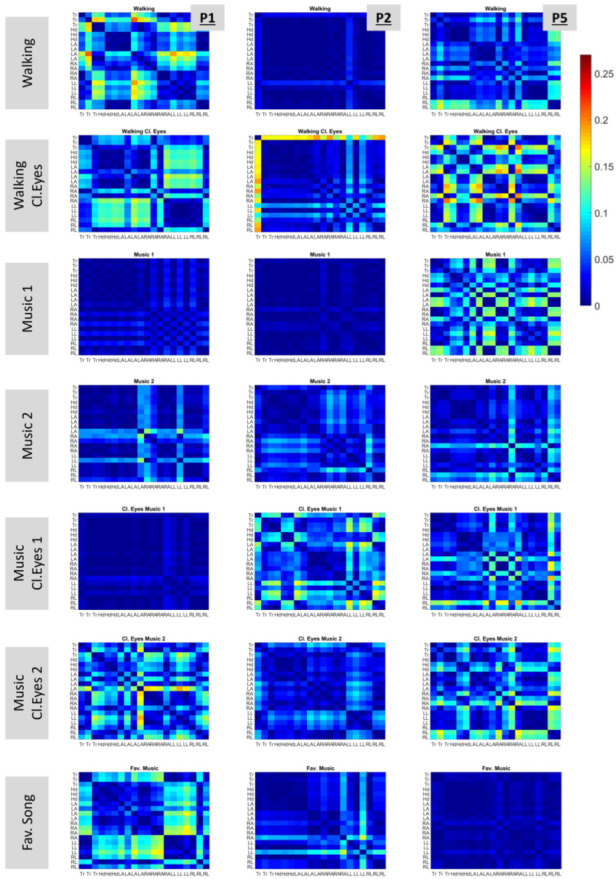
Differences in the slopes among body parts. Columns and rows represent different body parts. The first three correspond to data originating from the torso (Tr), the next three from the head (Hd), the next four from the left arm (LA), the next four from the right arm (RA), the next three from the left leg (LL), and the last three from the right leg (RL). The exact locations of body parts are shown in Figure 2B. Each cell color maps the difference between the slopes of the two corresponding body parts. The presented plots show data from three participants, namely, P1, P2, and P5.

To achieve this, matrices were built that display (in color) the absolute values of slope differences among all possible pairs of body parts. The matrices are shown in Figure 8 for P1, P2, and P5. These participants were indicatively chosen, as they had increased variability in slope differences among the conditions. For example, P1 demonstrated low slope differences for conditions “Music 1”, “Music 2”, and “Music Cl.Eyes 1” (the dark blue color dominates the graph) but high slope-difference variability for the rest of the conditions. Nevertheless, no specific condition with systematically low or high slope-difference variability applicable to all participants was indicated.

In these plots, it is interesting to observe the color patterns that point out similar slope differences in the kinematic chain. One instance is the “Walking Cl. Eyes” condition of P1, which has increased slope differences for the upper torso and head (columns) when compared with the left and right legs (rows), as indicated by the cyan color on the graph. The same pattern appears for the same participant in condition “Fav. Song”.

The outcomes of the acceleration data are displayed in Supplementary Figure S2. It is important to note that visual patterns observed in the speed figures do not necessarily translate to the acceleration figures.

### 3.3. Expanding Investigations to the Four Moments of the Gamma Distribution

**Figure 9:**
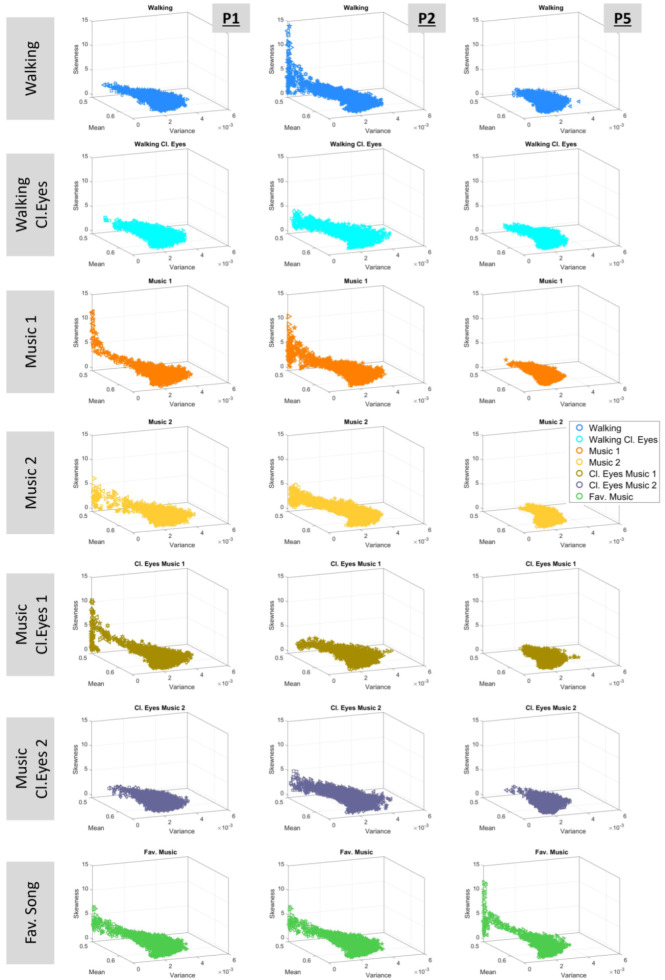
Estimated Gamma moments. The mean, μ, corresponds to the x-axis; the variance, σ, corresponds to the y-axis; the skewness corresponds to the z-axis; and the kurtosis is represented by the size of the marker.

A different perspective of the Gamma signatures can be gained by observing the four-moment plots, which are shown in Figure 9. The connection between the Gamma and the four-moment planes lies in the fact that the mean is equal to μ=ab and variance is $\sigma =ab^{2}$, where a denotes the shape and b is the scale. Skewness and kurtosis are estimated accordingly.

The Kruskal–Wallis test was applied to the four-moment parameters of each participant, indicating statistical significance among the experimental conditions. The outcomes are demonstrated in Table 4 and report the variance in the motor fluctuations of the walking task across all conditions, profiling the effects of sensory and contextual conditions.

The four moments of acceleration data are displayed in Supplementary Figure S3, and the results of the Kruskal–Wallis test are presented in Supplementary Table S2.

### 3.4. Exploring the Cumulative Space

**Figure 10:**
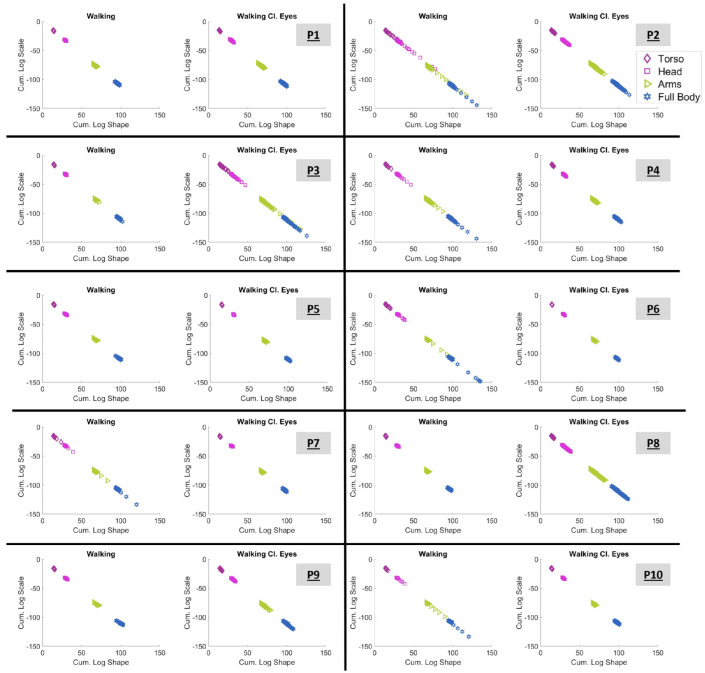
Cumulative log-shape and log-scale automatically self-clusters the kinematic chains of the torso (1 to 3), head (1 to 6), arms (1 to 14), and full body (1 to 20). The exact grouping of body parts is shown in Figure 2.

The value of the cumulative results, as explained in Section 2.4.7, can be seen in Figure 10 for all participants. The figure demonstrates the cumulative log–log shape and scale parameters of the 182 blocks of four kinematic chains, namely, the hip-to-torso chain (from sensors 1 to 3, as shown in Figure 2B), the hip-to-head chain (from sensors 1 to 6), the hip-to-arms chain (from sensors 1 to 14), and finally, the full-body chain (from sensors 1 to 20).

It is vital to appreciate that Figure 10 exhibits graphs of clearly clustered signatures. These clusters are defined by the kinematic chain to which they belong. And the outcome is impressively clear.

Next, the slope of the cumulative log–log Gamma plane and mean error (delta, Δ) from the fitting line was extracted. These two parameters along with the quadrant participation ratio enabled the creation of two new parameter spaces, as follows: (1) the plane spanned by the cumulative log–log Gamma slope vs. the mean delta (δ) from the fitting line and (2) the plane spanned by the LUQ/RLQ ratio vs. the slope of the cumulative log–log Gamma line.

#### 3.4.1. Choosing Sensory Preferability Through Parameter Space 1

**Figure 11:**
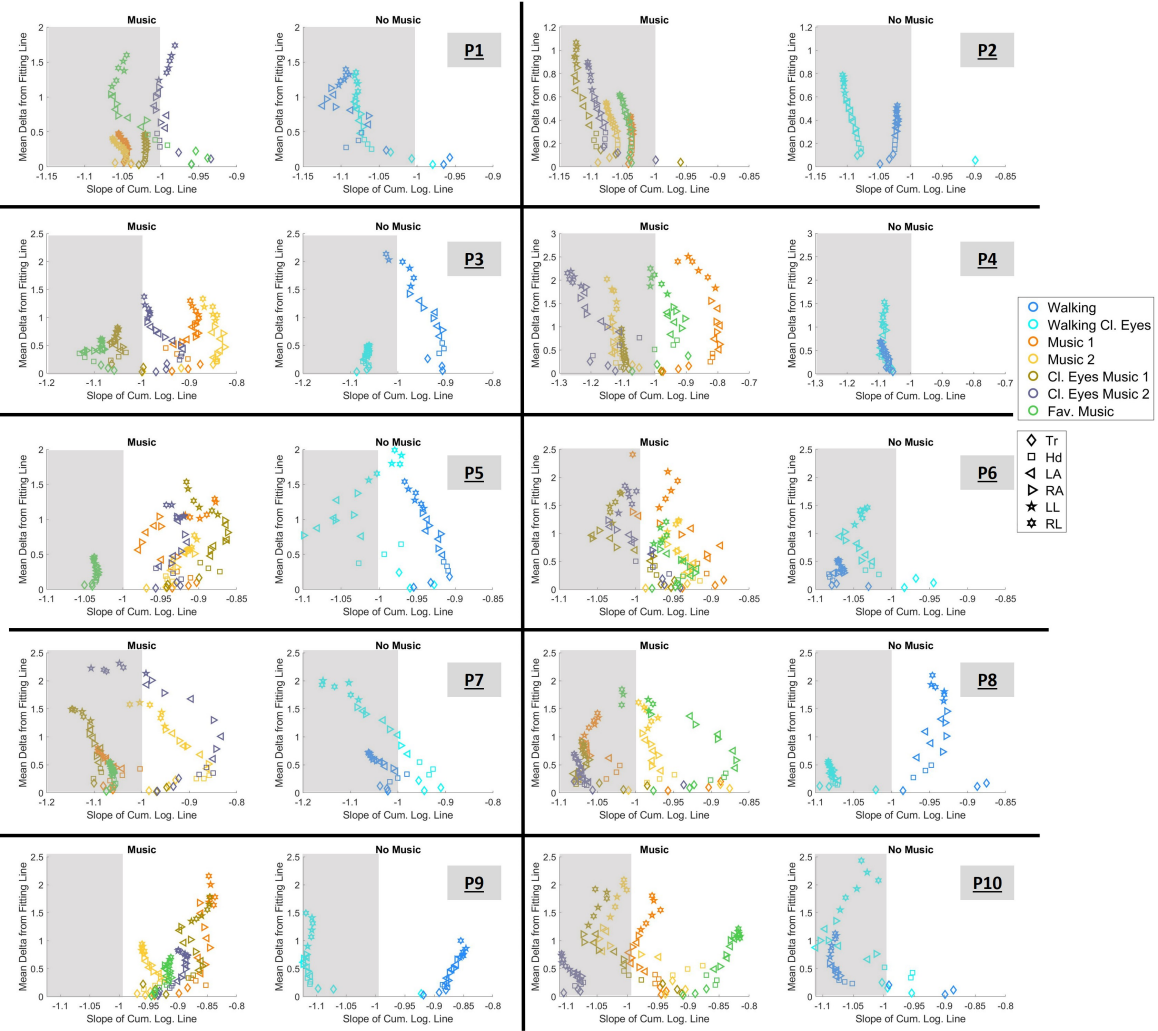
Parameter space defined by the cumulative logarithmic Gamma slope on the x-axis and the mean delta (δ) from the fitting line on the y-axis. Markers are color-mapped based on the condition and their shape indicates the body part they belong to. The shape of the marker represents the body part they belong to. The shaded area demonstrates the area where the absolute value of the slope is larger than 1.

In Figure 11, we can observe the data of each participant separated into two graphs. The right graph showcases conditions with music and the left graph shows conditions with no music. Each marker is color-mapped based on the condition it belongs to and the marker shape indicates the body part. Results from all 20 body parts are presented. We should recall here that each estimated point encompasses hundreds of measurements of fluctuations in the amplitude of linear speed peaks and spans an empirically estimated Gamma PDF family (from the overlapping block of data; see Section 2.4.3).

As explained in Section 2.4.6, slopes with absolute values larger than 1 indicate a faster rate of change toward the LRQ, demonstrating a better sensorimotor response. Thus, we are looking for conditions that lie on the left side of −1; the farther left the signatures of a condition are located, the better. This region is demonstrated in the graph by the gray-shaded area.

Figure 11 reveals that each experimental sensory context is separable and provides information about the preferred condition(s). Thus, when listening to music, participants performed favorably during the conditions:

- P1: “Music 1”, “Music 2”, “Music Cl. Eyes 1”, and “Fav. music”
- P2: for all conditions
- P3: “Music Cl. Eyes 1” and “Fav. music”
- P4: “Music 2”, “Music Cl. Eyes 1”, and “Music Cl. Eyes 2”
- P5: “Fav. music”
- P6: “Cl. eyes 1” and “Cl. eyes 2”
- P7: “Music 1”, “Music Cl. Eyes 1”, and “Fav. music”
- P8: “Music 1”, “Cl. eyes 1”, “Cl. eyes 2”
- P9: for no condition
- P10: “Music 1”, “Cl. eyes 2”, and “Cl. eyes 2”.

When the task was executed without listening to music, P1, P2, P4, P7, and P10 performed favorably during both conditions. However, the desirable outcome is noted in P5, P8, and P9 during the “Walking Cl.eyes” condition. In contrast, P1, P6, P7, and P10 performed best when simply “Walking” with open eyes. P4 was the only participant who seemed to perform similarly well in both conditions, yet the expansion of the mean delta value led us to choose “Walking” as the best condition.

Moreover, Table 5 displays the findings of the Wilcoxon rank-sum test for equal medians. The test was applied to the slope values of the conditions that included music versus the condition with no music (line 1) and the conditions where walking was conducted with open eyes versus closed eyes (line 2), indicating statistical significance.

The results of parameter space (1) for the acceleration data are shown in Supplementary Figure S4. In these graphs, it is important to note that although variability in the signatures exists (compared to the outcomes of speed data), there is at least one condition that satisfies the criteria in both data types (speed and acceleration).

#### 3.4.2. Choosing Sensory Preferability Through Parameter Space 2

**Figure 12:**
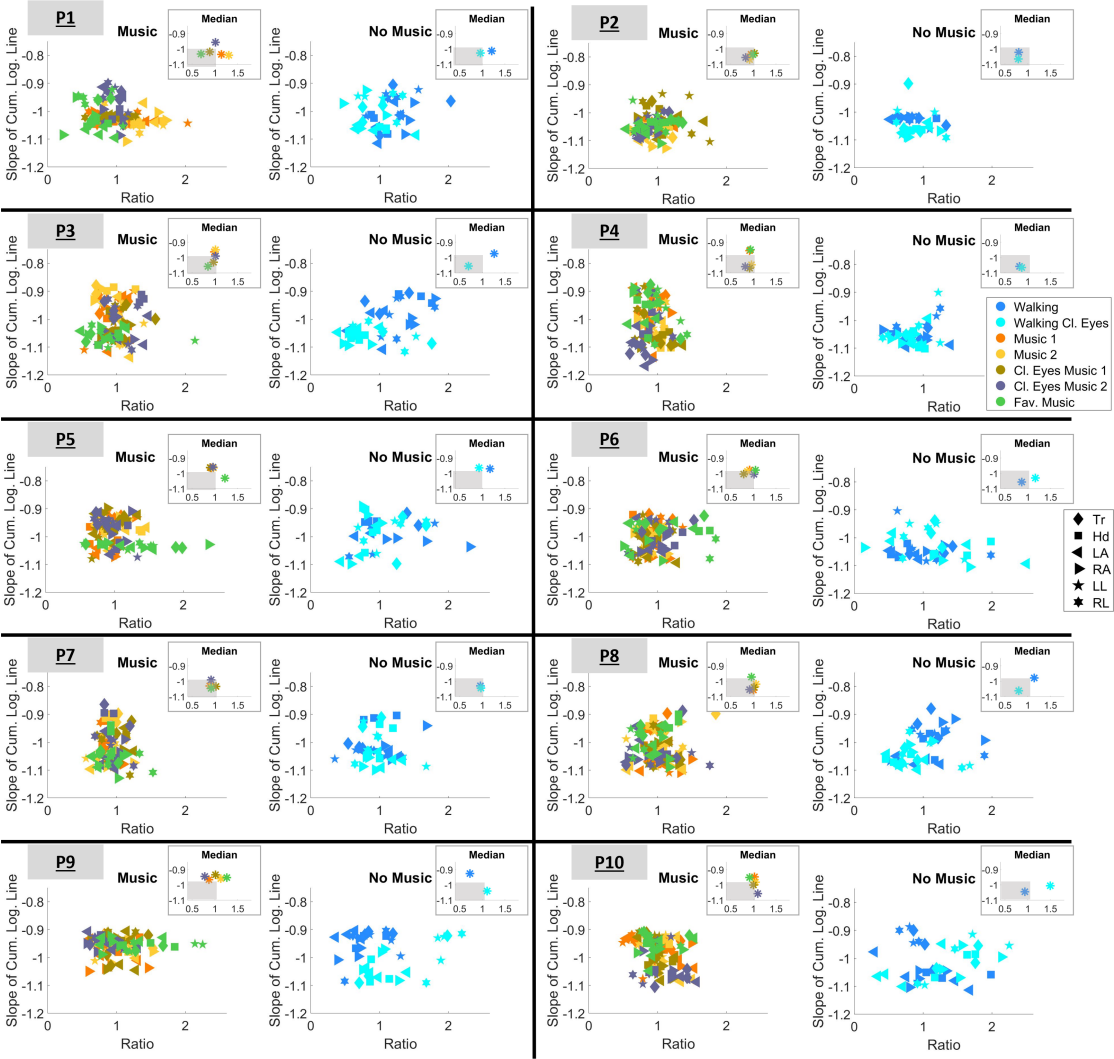
Parameter space defined by the LUQ/RLQ ratio and the slope of the cumulative logarithmic Gamma line. Markers are color-mapped based on the condition and the shape of the marker represents the body part. Insets demonstrate the median ratio and slope values of each condition. The gray-shaded area visualizes the region within which the slope and ratio criteria are satisfied.

The results concerning the parameter space (2) are presented in Figure 12. As previously explained in Section 2.4.4, a ratio that is less than 1 and closer to 0 expresses higher predictability. Therefore, here, we are looking for conditions whose signatures satisfy two criteria, i.e., (a) ratio<1 and (b) |slope|>1. To gain a clearer understanding of this, the median ratio and slope values are estimated for each condition. The results are shown in the insets of Figure 12. The gray-shaded area in the inset visualizes the region within which the two criteria are satisfied. As a result, it is now easier to determine the outcomes for each participant.

As such, when listening to music, participants perform best in the following conditions:

- P1: during condition “Fav. music”
- P2: during condition “Cl. eyes 2”
- P3: during condition “Fav. music”
- P4: during condition “Cl. eyes 2”
- P6: during condition “Cl. eyes 1”
- P7: during condition “Fav. music”
- P8: during condition “Cl. eyes 2”.

When the task was executed without listening to music, P1, P2, P3, P7, and P8 performed best during the “Walking Cl.eyes” condition. In contrast, P4, P6, and P10 performed best when simply “Walking” with open eyes. It is also worth noting that P5 and P9 did not satisfy either of the criteria simultaneously for any of the conditions and that P2 was the only participant who satisfied both criteria during all conditions.

The corresponding outcomes of the acceleration data are shown in Supplementary Figure S5. It is important to note that although differentiation exists among the scatters of signatures, some conditions satisfy the criteria for both data types (speed and acceleration). The results of the Wilcoxon rank-sum test are displayed in Supplementary Table S3, indicating that both ratio and slope have statistical significance when comparing the conditions that have music against the conditions that have no music.

## 4. Discussion and Possible Applications

This study introduces novel methods that enable the extraction of the preferred sensory input from the motor stream. Using an experimental paradigm, where walking served as the consistent motor task across all conditions, we were able to detect the different sensory information collected by the motor stream, as explained by the principle of reafference (see Section 1.1). Furthermore, we were able to distinguish the audio and visual conditions that encouraged the sensorimotor system to perform better.

### 4.1. Metrics, Parameter Space, and Findings

Variant metrics are presented in this paper and each one played a key role in interpreting the findings. The plane described by the shape and scale parameters of the Gamma family of distributions was used to estimate the Euclidean distance between the two extreme signatures, and it was more elaborately studied in the four-dimensional graph of the four moments (i.e., mean, variance, skewness, and kurtosis). New informative parameter spaces were discovered by the accumulation of the shape and scale parameters, as displayed in Figure 10. Next, the extraction of the slope and the quadrant participation ratio, along with the Euclidean distance, were used as criteria to evaluate the performance of each participant.

Regarding the **four moments**, the findings showed that—for all participants—the Gamma stochastic signatures, described by the mean, variance, skewness, and kurtosis of all conditions, have statistical significance (Table 4 and Supplementary Table S2). This indicates that, first, the presented methods can capture the very small fluctuations in movement that differentiate the walking patterns among conditions, and second, the variability of these fluctuations is significant from condition to condition. These findings highlight the methods’ ability to detect subtle movement fluctuations that differentiate walking patterns among conditions and reveal significant variability in these fluctuations.

The **cumulative space** created through the accumulation of shape and scale parameters revealed compelling patterns. Figure 10 demonstrates the connections between the stochastic signatures of body parts that belong to the same kinematic chain, since the clusters that were created are clearly shifted. The longer the kinematic chain, the lower and to the right the signature will lie. Of course, the kinematic chain creates relationships between body parts, and the closer the body parts are, the more they will be affected by each other’s motion. This discovery encourages further exploration of metrics and tools within this cumulative space.

To evaluate the performance of each participant during a condition, the following three different metrics were used as criteria: the Euclidean distance, the slope of the cumulative logarithmic Gamma line, and the quadrant participation ratio.

The **Euclidean distance** between the two extremes of the logarithmic Gamma line indicates a shift in the shape and scale parameters between the two extreme cases (from most noisy to least noisy window). As such, the longer the line, the larger the effort of the nervous system to learn and adapt. Figure 7B reveals the statistical significance among the conditions.

The **slopes** of the cumulative logarithmic Gamma lines when plotted in parameter space (1) formed self-clustered scatter plots that clearly distinguished different conditions. Given that |slope| > 1 indicates a faster rate of change toward the LRQ, it enables the identification of preferable sensory conditions (Figure 11 and Supplementary Figure S5). It is also important to note that there is at least one condition that satisfies the criteria in both data types, i.e., speed and acceleration. The rank-sum test applied to the slope values (Table 5) indicated statistical significance when comparing the music versus no-music conditions and the open-eyes versus closed-eyes conditions. However, significance was only detected for acceleration data in music versus no-music comparisons (Supplementary Table S3).

The LUQ/RLQ **ratio** was also employed along with slope in parameter space (2) and had to be ratio<1. Parameter space (2) was more restrictive since two parameters (ratio and slope) had to be satisfied (Figure 12 and Supplementary Figure S4). Based on the rank-sum test displayed in Table 5, the ratio value of the speed data did not have statistical significance. With the acceleration data, the rank-sum test distinguished significance when comparing the music versus no-music conditions (Supplementary Table S3) but not when comparing the open-eyes versus closed-eyes conditions. Therefore, the ratio parameter seems to be the most useful for graphical representations.

Rank-sum tests for ratio values provided limited insights. However, the rank-sum outcomes of the slope values enabled the identification of key differences between walking with open vs. closed eyes—conditions that activate the participant’s kinesthetic sense of proprioception—and walking in the presence of music or silence; see Table 5 and Supplementary Table S3.

The inclusion of the **favorite song** condition aimed to examine whether participants’ preferences correlated with sensory favorability or sensorimotor “joy”. Parameter space(2) revealed that for only 4 out of 10 participants (P1, P2, P3, and P7), the choice of a favorite song benefited walking performance, or 4 out of 7 who satisfied both criteria. Surprisingly, the conditions of “Walking Cl. Eyes 1” and “Walking Cl. Eyes 2” satisfied both criteria for 7 out of 10 participants (P1, P2, P3, P4, P6, P7, and P8), indicating that walking with closed eyes while listening to music may not be a comfortable task; however, the lack of vision may require higher predictability from the motor system in order to cope. Likewise, for conditions where no music was present, the bodily biorhythms from the control participant revealed a preference for the closed-eye condition. As previously noted, 5 out of 8 participants—satisfying the criteria—performed favorably when walking with closed eyes.

### 4.2. Key Elements of the Study Design

Some key design elements that influenced the outcomes of this study include the following: the consistent use of the same motor task across all conditions, the incorporation of the Gamma continuous process during the processing of the signal, and the simultaneous investigation of speed and acceleration data types.

The use of a consistent motor task, i.e., **walking**—across all conditions—was a key factor of the study. This uniformity served as the foundation that allowed us to argue that the presented methods enable the extraction of sensory and contextual information from the motor stream, thereby relating the findings to the principle of reafference. Since the motor task remains the same, the variability in outcomes can be attributed to the sensory perceptions of differing conditions, as reflected in the reafferent signals captured by the motion-recording equipment. Indeed, in the future, it would be interesting to expand the research of the methods to additional sensory modalities (e.g., smell—employing aromatherapy diffusion) and varying sensory parameters (e.g., volume or color of the lighting in a room).

Another critical element is that although the data used in the study were pre-recorded, the analysis pipeline was designed in a way that could facilitate **real-time** applications as well. This was achieved via the incorporation of the Gamma continuous process under the general rubric of Poisson random processes (Section 2.4.3). A 5 s data window was used for analysis, but future studies could explore other buffering durations, based on the sampling rate of the equipment used and the needs of the future study.

Another key point of this work is that it investigates, in parallel, the findings from both the **linear speed and acceleration** streams to examine further possibilities for information retrieval. As speed describes the rate of change of position and acceleration describes the rate of change of speed, analyzing both provides insights into different dynamics of movement simultaneously. As already noted for the last two criteria presented in Section 3.4.1 and Section 3.4.2, there are differences between the outcomes of the two data types. For instance, if we compare the results of P1 in Figure 11 and Supplementary Figure S5, we will observe that conditions are not distributed in similar order. Condition “Music Cl. Eyes 2” has slope values, |slope|≤1, in the case of speed data, and |slope|≥1.11 in the case of acceleration data. Despite this, performance is the best for both data types (speed and acceleration) during condition “Music 2”. Additionally, it can be noted that there is at least one condition that satisfies the criteria in both data types for the rest of the participants as well.

Lastly, it is vital to mention that the speed and acceleration data types used for this study could be collected from various motion-recording **equipment**, including wearables and IMUs, which register acceleration. This flexibility means that the methods are not confined to the use of a motion-capture system (which registers position data that were used to estimate speed and acceleration). Instead, they can also be applied to low-cost sensors that allow the direct extraction of speed or acceleration data. While some could be concerned about the amount of noise a low-cost sensor registers along with the motor steam, prior studies, i.e., Refs. [20,28], have demonstrated that using less expensive wearable technology along with the MMS structure led to valuable findings. This highlights the robustness and adaptability of the methods for different settings and technologies.

### 4.3. Affecting Temporal and Spatial Features of the Motor Stream

An earlier study [30] conducted on the same dataset (as this study) also revealed the integration of other sensory information within the motor stream. The study analyzed the data collected from all conditions where walking was executed with open eyes while listening to music and in silence (conditions 1, 3, 4, and 7). The speed data and the heart activity (ECG) were tracked. The findings indicate that different forms of music may affect the **temporal features** of linear speed and heart rate variability, while they do not consistently impact the amplitude of the signals (spatial feature). Specifically, according to the Kolmogorov–Smirnov test, the inter-peak intervals of MMS timings were highly sensitive to the changes in music, but no statistical significance was found when examining the amplitude of the speed MMS amplitude. These results were attributed to bodily signal entrainment in response to music.

In contrast, the current study presents findings that highlight the significance of **spatial features** in the motor stream. By analyzing the metrics of the four moments (mean, variance, skewness, and kurtosis), statistical significance was observed in the spatial characteristics of speed and acceleration MMS. Although a prior study [30] did not detect amplitude-based fluctuations in the speed waveform directly, the four-moment analysis in this research uncovered additional insights regarding the spatial properties of the motor stream.

The emphasis on temporal features in [30] was further investigated in [20], which presented a co-adaptive closed-loop interface driven by audio augmented with the dancer’s heart rate in real-time. In this setup, the heart rate of the dancer was mapped to the speed of the salsa song performed, requiring continuous adaptation of movements to the dynamically changing rhythm. Unlike the stable rhythmic input provided by walking with music in [30], this real-time adaptive setup did not impact the temporal features of bodily signals, as it did not create stable rhythmical audio. However, it revealed a significant shift in the stochastic signatures of the **spatial features** of bodily MMS, offering new insights into the dynamics of movement and sensory integration.

### 4.4. Theory and Possible Applications

The findings of this study, specifically the ability to extract sensory preferability from motor streams, align with modern research on **contemporary models of sensorimotor integration**. Wolpert and Flanagan [36] discussed how predictive control mechanisms rely on sensory feedback and efference copies to refine motor commands. The findings in this study provide further empirical support for such theories, demonstrating how the Gamma plane’s statistical properties capture sensory–motor interactions.

Also, studies on motor adaptation have emphasized the importance of **variability in movement learning**. Herzfeld et al. [37] explored how the nervous system maintains a memory of errors to fine-tune motor actions over time. Similarly, Dhawale et al. [38] described variability as an adaptive strategy in sensorimotor tasks, suggesting that a well-tuned nervous system balances stability and flexibility. These insights further validate our approach, where the LUQ/RLQ ratio and the Euclidean distance of extreme Gamma signatures serve as indicators of a system’s predictive capacity.

In the more applicable side of research, the methods presented here could enrich **co-adaptive interfaces**, as described in [17,18], dedicated to monitoring bodily signals for further evaluation of the motor stream and adaptation to the personalized preferences of the user. They could also inform research, similar to studies such as [39], which explored wearable sensor-based monitoring systems for real-time motion analysis. In particular, adaptive rehabilitation systems could be aided by integrating real-time motor stream analysis, optimizing interventions based on individual sensory preferability. Moreover, interfaces designed for biofeedback applications, such as ECG-based auditory augmentation [39], could be extended to include motor stream variability as an additional input parameter. Other **real-time interfaces**, such as [19], designed to augment features of the ECG signal through auditory feedback, could benefit. The significance is that by registering the motor stream, it becomes possible to study the perception of the PNS as captured by the motion-recording systems when the interface augments other bodily signals, such as ECG and temperature.

In the context of **scientific research and clinical practices**, the presented data types and methods provide tools to study sensory–motor systems upon experimental and clinical interventions, as seen in [15,16]. Specifically, advancements in interactive and rehabilitation technologies, such as exoskeletons and prosthetics, could benefit from a deeper understanding of sensory preferability in motor control. Recent findings in motion sensor technology suggest that the adaptive calibration of wearable devices, based on an individual’s sensorimotor responses, can significantly improve user experience and rehabilitation outcomes [40].

Lastly, the current design of the methods facilitates (1) the capability of transferring the methods in a real-time setup and (2) the possible utilization of a wide range of equipment along with the methods simplifying the process of incorporating the presented methods in an existing interface. As a result, this study’s design presents a strong case for further research into sensory–motor integration using motor stream analysis. Future work should explore its applicability in real-time adaptive systems, human–machine interactions, and clinical rehabilitation frameworks.

## 5. Conclusions

In this study, participants performed a specific motor task—walking—under two distinct visual and auditory conditions. By manipulating these sensory parameters within the same motor task, we developed computational methods capable of isolating sensory information embedded within motor streams recorded by a motion-capture system, a phenomenon grounded in the “principle of reafference”.

By leveraging the MMS structure to process linear speed and acceleration data through the continuous Gamma process, we successfully identified stochastic signatures and characterized their four statistical moments. Our analysis demonstrated statistically significant distinctions across conditions, as evidenced by metrics such as the Euclidean distance of extreme signatures and the four-moment statistical measures. Furthermore, we expanded the parameter space by systematically mapping the accumulation of the Gamma plane’s shape and scale parameters. Notably, two key metrics—the slope of the cumulative logarithmic Gamma line and the LUQ/RLQ ratio—enabled robust self-clustering of data based on sensory conditions. This breakthrough allowed us to detect each individual’s personalized preferred sensory condition, identifying the specific sensory input that optimizes motor system performance by minimizing noise and enhancing predictability.

In conclusion, this work presents a platform for objective and automated analysis of the afferent kinesthetic stream extracted from motor outputs. This platform holds potential applications in interactive interfaces, laboratory research, and clinical settings.

## Figures and Tables

**Table 1:** Condition, description, and duration.

Condition	Description	Duration
# 1	Walking	5 min
# 2	Walking Cl. Eyes	5 min
	**Walking Open Eyes & Music**	5 min
# 3	Music 1	2.17 min
# 4	Music 2	2.43 min
	**Walking Closed Eyes & Music**	5 min
# 5	Music Cl. Eyes 1	2.17 min
# 6	Music Cl. Eyes 2	2.43 min
# 7	Fav. Music	5 min

**Table 2:** Euclidean distance between the two extreme logarithmic Gama signatures presented in Figure 6. Highlighted in red are the values below 2 and highlighted in blue are the values above 10.

	Walking	Cl.Eyes	Music 1	Music 2	M.Cl.E.1	M.Cl.E.2	Fav.M.	Mean
P1	2.6153	3.0803	8.3027	9.9585	16.2696	2.5463	3.6650	6.63
P2	15.4041	5.0433	9.8127	7.5092	3.8996	6.1839	7.9351	7.96
P3	3.0795	9.9164	3.4311	2.2010	4.0664	2.7714	10.8285	5.18
P4	6.5625	3.3152	1.8821	1.3742	4.5432	1.8562	2.3375	3.12
P5	1.7314	2.1357	1.5971	1.5458	1.7745	1.9206	12.8226	3.36
P6	7.1734	2.5319	2.2215	1.3054	2.2047	1.7081	1.9690	2.73
P7	7.9360	1.6930	6.1193	2.3235	3.3887	2.1550	8.3185	4.56
P8	3.1501	6.2031	3.1024	4.2011	5.1410	6.3765	1.6493	4.26
P9	1.9820	4.3195	2.5957	1.4136	2.2465	1.5484	1.5303	2.23
P10	5.1849	4.2032	2.9588	2.3339	4.1045	6.4769	1.7299	3.86

**Table 3:** *p*-Values of the Kruskal–Wallis test applied to the Euclidean distance findings. The graphs of the test are presented in Figure 7.

**KW Test**	**P1**	**P2**	**P3**	**P4**	**P5**
*p*-values	3.78 $\times \;10^{-17}$	1.08 $\times \;10^{-174}$	4.95 $\times \;10^{-16}$	1.29 $\times \;10^{-15}$	3.59 $\times \;10^{-13}$
	**P6**	**P7**	**P8**	**P9**	**P10**
*p*-values	2.25 $\times \;10^{-10}$	2.54 $\times \;10^{-11}$	1.59 $\times \;10^{-6}$	4.39 $\times \;10^{-6}$	6.99 $\times \;10^{-7}$

**Table 4:** Statistical significance of the four moments, namely, mean, variance, skewness, and kurtosis, across all conditions. The non-parametric one-way ANOVA-Kruskal–Wallis test was employed to differentiate the general effects of experimental conditions (columns) over body parts (rows). The test was applied to each participant separately.

**KW Test**	**P1**	**P2**	**P3**	**P4**	**P5**
Mean	8.02 $\times \;10^{-254}$	8.12 $\times \;10^{-174}$	1.64 $\times \;10^{-251}$	0	1.98 $\times \;10^{-171}$
Variance	2.79 $\times \;10^{-92}$	1.33 $\times \;10^{-179}$	4.86 $\times \;10^{-177}$	8.6 $\times \;10^{-80}$	2.60 $\times \;10^{-60}$
Skewness	7.81 $\times \;10^{-213}$	6.18 $\times \;10^{-166}$	2.48 $\times \;10^{-219}$	0	2.62 $\times \;10^{-201}$
Kurtosis	5.20 $\times \;10^{-41}$	2.56 $\times \;10^{-86}$	6.61 $\times \;10^{-139}$	1.38 $\times \;10^{-72}$	1.35 $\times \;10^{-64}$
	**P6**	**P7**	**P8**	**P9**	**P10**
Mean	0	2.29 $\times \;10^{-94}$	0	0	0
Variance	4.05 $\times \;10^{-23}$	2.09 $\times \;10^{-47}$	1.73 $\times \;10^{-198}$	1.88 $\times \;10^{-66}$	2.88 $\times \;10^{-18}$
Skewness	0	7.97 $\times \;10^{-89}$	0	0	0
Kurtosis	6.83 $\times \;10^{-70}$	1.33 $\times \;10^{-33}$	4.82 $\times \;10^{-71}$	3.59 $\times \;10^{-101}$	3.64 $\times \;10^{-86}$

**Table 5:** Wilcoxon rank-sum test applied to the ratio and slope values of the conditions that included music versus the condition with no music (line 1) and the conditions where walking was conducted with open eyes versus closed eyes (line 2).

Wilcoxon Rank-Sum Test	Ratio	Cum. Log. Gamma Slope
Music vs. No Music	>0.01	$1.26\;\times \;10^{-27}$
Op.Eyes vs. Cl.Eyes	>0.01	$4.03\;\times \;10^{-5}$

## Data Availability

Data sharing is not applicable due to privacy.

## Supplementary Materials

The following supporting information can be downloaded at https://www.mdpi.com/article/10.3390/s25072087/s1. Table S1: Euclidean distance between the two extreme Log Gama signature presented in Supplementary Figure S1. Highlighted in red color are the values below 2 and highlighted in blue are the values above 10. Table S2: Statistical Significance of four moments: mean, variance, skewness, and kurtosis, across all conditions. The non-parametric one-way ANOVA-Kruskal Wallis test was employed to differentiate general effects of experimental conditions (columns) over body parts (rows). The test was applied to each participant separately. Table S3: Wilcoxon rank-sum test applied on the ratio and slope values of the conditions that include music versus the condition with no music (line 1) and the conditions where walking was done with open eyes versus closed eyes (line 2). Figure S1: Log-log representation of the shape and scale parameters of the Gamma Distribution for the conditions “Walking” and “Walking with closed eyes”. Demonstrated plots correspond to all participants, from P1 to P10. The color-mapped markers correspond to different body-parts (grouped as presented in Figure 2 of main text). Tr stands for torso; Hd for head; LA for left arm; RA for right arm; LL for left leg; and RL for right leg. The insets highlight the PDFs of the presented Gamma signatures. The X axis of the inset represents MMS (the normalized peaks of the speed) and the Y axis represents Probability Density. Figure S2: Differences of the slopes among body-parts. Columns and rows represent different body-parts. The first 3 correspond to data originated from the torso (Tr), the next 3 from the head (Hd), the next 4 from the left-arm (LA), the next 4 from the right-arm (RA), the next 3 from the left-leg (LL), and the last 3 from the right-leg (RL). The exact location of them is demonstrated in Figure 3B of main text. Each cell colormaps the difference of the slopes of the two corresponding body-parts. The presented plots show data from three participants, P1, P2, and P5. Figure S3: Estimated Gamma moments. The mean, μ, corresponds to the x-axis; the variance, σ, corresponds to the y-axis; the skewness corresponds to the z-axis; and the kurtosis is represented by the size of the marker. Figure S4: Parameter space defined by the cumulative logarithmic Gamma slope on the x-axis and the Mean Delta (δ) from the fitting line on the y-axis. Markers are color-mapped based on the condition and their shape indicated the body. The shape of the marker represents the body-part they belong to. The shaded area demonstrates the area where the absolute value of the slope is bigger than 1. Figure S5: Parameter space defined by the LUQ/RLQ ratio and the slope of the cumulative logarithmic Gamma line. Markers are color-mapped based on the condition and the shape of the marker represents the body-part. Insets demonstrate the median ratio and slope values of each condition.
