# Supplementary material for: Extracting Sensory Preferability from Motor Streams
Source: Sensors (Basel). 2025 Mar 26;25(7):2087. doi: 10.3390/s25072087 (PMC11991022; doi:10.3390/s25072087)
Supplement: Supplementary file 1 [file sensors-25-02087-s001.zip › sensors-3499257-supplementary.pdf]

Article

Extracting Sensory Preferability from Motor Stream

Vilelmini Kalampratsidou

Department of Product and System Design, Aegean University, 84100 Ermoupolis, Syros, Cyclades, Greece; vilelmini.kalabratsidou@gmail.com

1. Supplementary Material

All figures presented in "Supplementary Material" demonstrate the results of the linear acceleration data analysis, while following the exact analysis steps presented in "Main Documents". Therefore, the figures presented here are aligned with the "Main Documents" figures, displaying the outcome of studying the same parameters but on different types of motor streams.

Table S1. Euclidean distance between the two extreme Log Gama signature presented in Supplemen-tary Figure S1. Highlighted in red color are the values below 2 and highlighted in blue are the values above 10.

	Walking	Cl.Eyes	Music 1	Music 2	M.Cl.E.1	M.Cl.E.2	Fav.M.
P1	6.1158	6.4324	11.6744	10.9922	13.9312	4.2059	6.3803
P2	12.1679	9.1577	10.4756 3	8.1221	7.3859	7.8833	8.7794
P3	8.4373	7.7938	4.8801	9.2823	7.0254	7.8836	9.3746
P4	9.4450	6.3895	5.3152	2.3832	9.4781	5.8462	4.2002
P5	1.1768	1.3750	1.2517	1.1336	1.7298	1.5075	8.5675
P6	3.2025	1.8262	1.7851	1.5392	1.7192	1.5744	1.2071
P7	6.0857	5.1553	7.3959	4.3461	5.2864	5.7611	6.3904
P8	3.7881	9.1222	6.3292	3.6471	5.3593	8.3811	3.1185
P9	1.3216	1.5316	1.4631	1.3981	1.3547	1.4878	1.4615
P10	4.6438	2.9275	2.8472	1.4713	3.4398	3.5582	1.4464

Academic Editor: Giovanni Saggio

Received: 11 February 2025
Revised: 15 March 2025
Accepted: 19 March 2025
Published: 26 March 2025

Citation: Kalampratsidou, V. Extracting Sensory Preferability from Motor Stream. *Sensors* **2025**, *25*, 2087. https://doi.org/10.3390/s25072087

Copyright: © 2025 by the author. Licensee MDPI, Basel, Switzerland. This article is an open access article distributed under the terms and conditions of the Creative Commons Attribution (CC BY) license (https://creativecommons.org/licenses/by/4.0/).

Table S2. Statistical Significance of four moments: mean, variance, skewness, and kurtosis, across all conditions. The non-parametric one-way ANOVA-Kruskal Wallis test was employed to differentiate general effects of experimental conditions (columns) over body parts (rows). The test was applied to each participant separately.

**KW test**	P1	P2	P3	P4	P5
Mean	$1.17\ 10^{-78}$	$1.45\ 10^{-53}$	$5\ 10^{-106}$	0	0
Variance	$1.65\ 10^{-110}$	$3.48\ 10^{-149}$	$6.11\ 10^{-105}$	$7.36\ 10^{-125}$	0
Skewness	$3.44\ 10^{-89}$	$3.66\ 10^{-62}$	$5.421\ 10^{-108}$	$1.11\ 10^{-295}$	0
Kurtosis	$2.51\ 10^{-144}$	$1.66\ 10^{-107}$	$1.14\ 10^{-70}$	$9.41\ 10^{-211}$	0
	P6	P7	P8	P9	P10
Mean	$1.22\ 10^{-157}$	$4.84\ 10^{-66}$	$3.14\ 10^{-133}$	$1.94\ 10^{-136}$	$3.06\ 10^{-231}$
Variance	$2.63\ 10^{-76}$	$7.11\ 10^{-59}$	0	$5.37\ 10^{-58}$	$1.44\ 10^{-66}$
Skewness	$5.53\ 10^{-95}$	$5.80\ 10^{-60}$	$6.04\ 10^{-140}$	$2.74\ 10^{-123}$	$3.94\ 10^{-208}$
Kurtosis	$1.47\ 10^{-58}$	$9.73\ 10^{-55}$	$4.24\ 10^{-251}$	$1.35\ 10^{-72}$	$6.12\ 10^{-159}$

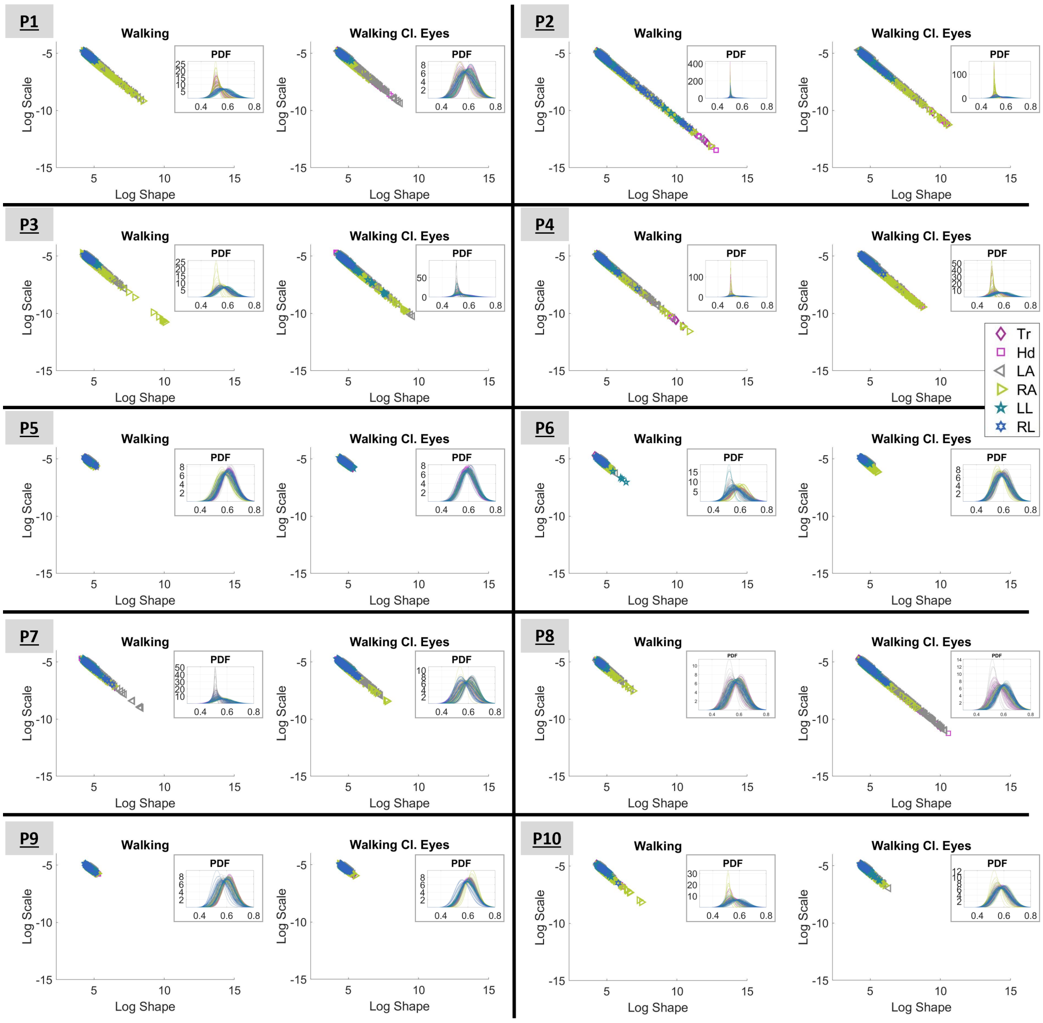

Figure S1. Log-log representation of the shape and scale parameters of the Gamma Distribution for the conditions "Walking" and "Walking with closed eyes". Demonstrated plots correspond to all participants, from P1 to P10. The color-mapped markers correspond to different body-parts (grouped as presented in Figure 2 of main text). Tr stands for torso; Hd for head; LA for left arm; RA for right arm; LL for left leg; and RL for right leg. The insets highlight the PDFs of the presented Gamma signatures. The X axis of the inset represents MMS (the normalized peaks of the speed) and the Y axis represents Probability Density.

Table S3. Wilcoxon rank-sum test applied on the ratio and slope values of the conditions that include music versus the condition with no music (line 1) and the conditions where walking was done with open eyes versus closed eyes (line 2).

**Wilcoxon rank sum test**	Ratio	Cum. Log. Gamma Slope
Music vs No Music	8.1110^{-4}	5.6010^{-48}
Op.Eyes vs Cl.Eyes	> 0.01	> 0.01

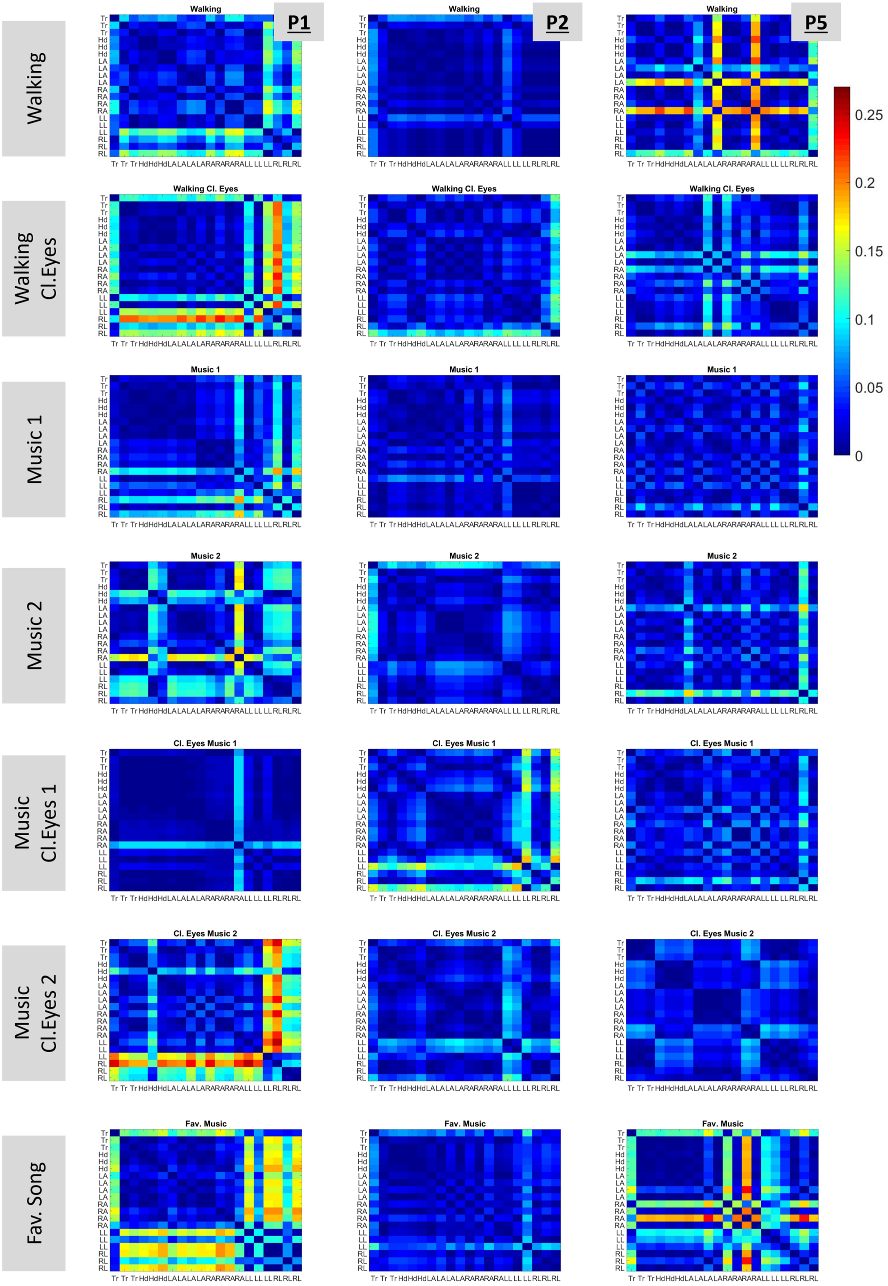

Figure S2. Differences of the slopes among body-parts. Columns and rows represent different body-parts. The first 3 correspond to data originated from the torso (Tr), the next 3 from the head (Hd), the next 4 from the left-arm (LA), the next 4 from the right-arm (RA), the next 3 from the left-leg (LL), and the last 3 from the right-leg (RL). The exact location of them is demonstrated in Figure 2.B of main text. Each cell colormaps the difference of the slopes of the two corresponding body-parts. The presented plots show data from three participants, P1, P2, and P5.

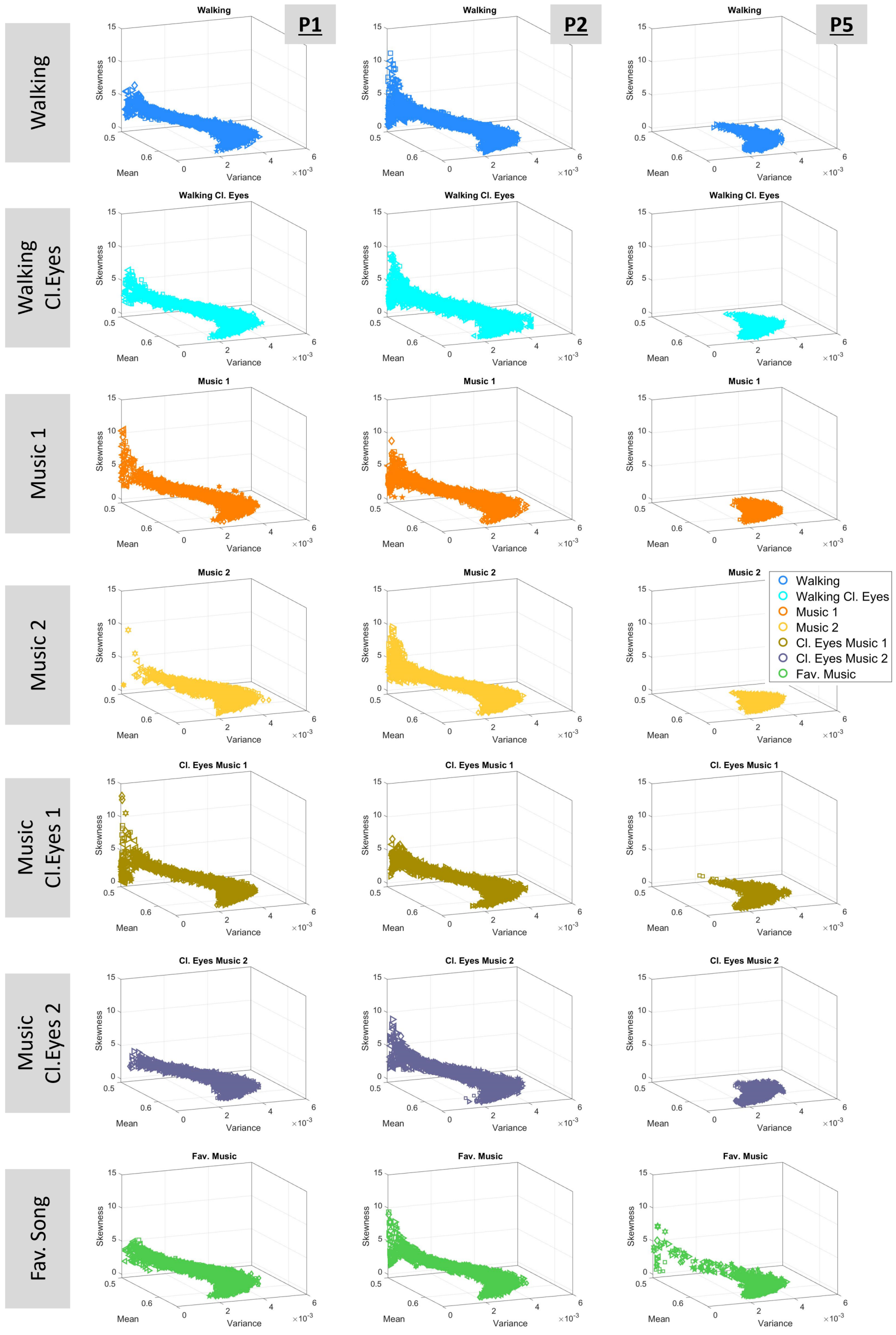

Figure S**3.** Estimated Gamma moments. The mean, μ, corresponds to the x-axis; the variance, σ, corresponds to the y-axis; the skewness corresponds to the z-axis; and the kurtosis is represented by the size of the marker.

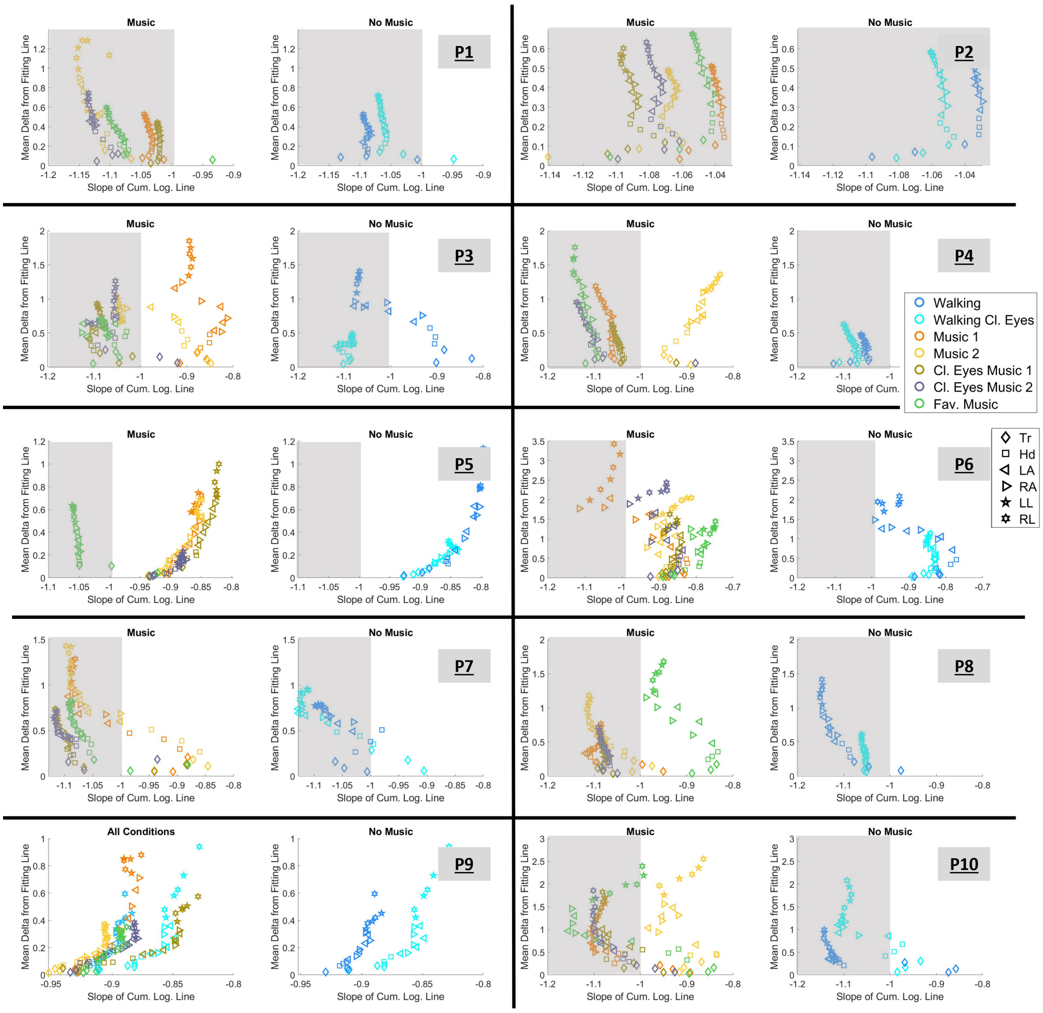

Figure S4. Parameter space defined by the cumulative logarithmic Gamma slope on the x-axis and the Mean Delta (δ) from the fitting line on the y-axis. Markers are color-mapped based on the condition and their shape indicated the body. The shape of the marker represents the body-part they belong to. The shaded area demonstrates the area where the absolute value of the slope is bigger than 1.

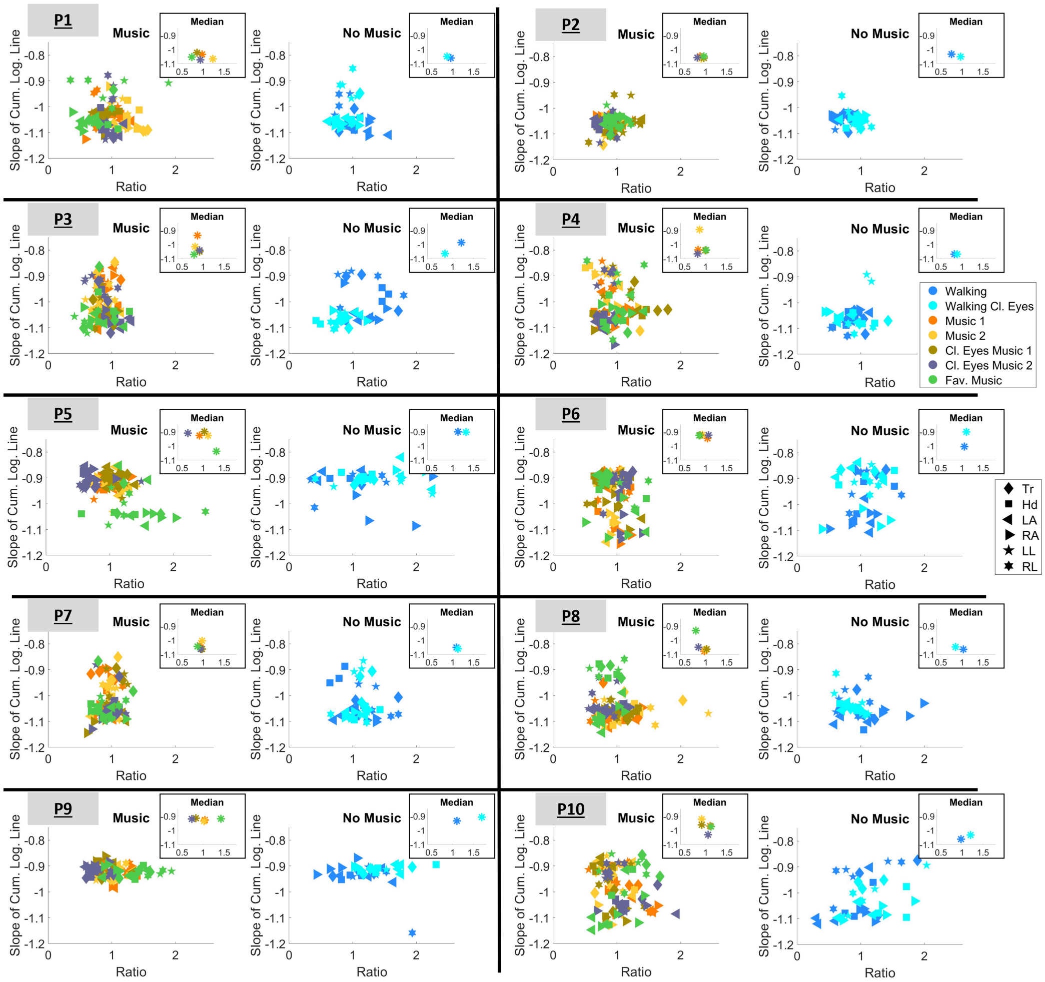

Figure S5. Parameter space defined by the *LUQ*/*RLQ* ratio and the slope of the cumulative logarithmic Gamma line. Markers are color-mapped based on the condition and the shape of the marker represents the body-part. Insets demonstrate the median ratio and slope values of each condition.
